# Perceptions and experiences of women with premature ovarian insufficiency about sexual health and reproductive health

**DOI:** 10.1186/s12905-021-01197-5

**Published:** 2021-02-08

**Authors:** Somayeh Moukhah, Behzad Ghorbani, Zahra Behboodi-Moghadam, Simin Zafardoust

**Affiliations:** 1 Department of Reproductive Health, School of Nursing and Midwifery, Tehran University of Medical Sciences, Tehran, Iran; 2 Reproductive Biotechnology Research Center, Avicenna Research Institute, ACECR, Tehran, Iran

**Keywords:** Primary ovarian insufficiency, Qualitative research, Reproductive health, Sexual health

## Abstract

**Background:**

Premature ovarian insufficiency (POI) is a condition with impaired ovarian function that occurred in women before the age of 40. Considering that women with POI are in reproductive age and their fertility and sexual life are afflicted by this disorder directly, the present study aimed to investigate perception and experience of women with POI of sexual and reproductive health (SRH).

**Methods:**

This is a qualitative that was implemented based on the conventional content analysis approach. The data were collected using semi-structured in-depth interviews with 16 women having POI, based on purposeful sampling and continued until data saturation. The participants were women with POI that referred to the three infertility center in Tehran, Iran. The audio recorded data were transcribed verbatim and then analyzed using conventional content analysis based on the method proposed by Zhang and Wildmouth.

**Results:**

After content analysis of the interviews with a focus on the perception and experience of women with POI of SRH, four main categories emerged i.e. endangerment of women's health, psychological agitation, disruption of social life and disturbance in sexual life.

**Conclusion:**

POI affects different aspects of women SRH (women physical, psychological, social and sexual heath). Therefore, knowledge of patients' concerns by health professionals is helpful to improve service delivery and increasing the effectiveness of treatment interventions by a comprehensive health care attitude.

## Background

Premature ovarian insufficiency (POI) is a condition with impaired ovarian function that occurred in women before the age of 40 [1] and is identified by oligo/amenorrhea for at least 4 months, and an elevated FSH level > 25 IU/I on two occasions 4 weeks apart [2, 3]. The prevalence of POI is different in several studies and it is depended on population characteristics such as racial, social, and economic factors, and lifestyle [2, 4]. For example, in a national register study in Sweden, prevalence of POI in women was 1.9% in 2018 [5] and another study reported 0.91% in Estonia [6]. Also some studies in Iran demonstrated 3.2–5.9% of women experience POI but, the global prevalence of POI was reported as 3.7% in a meta-analysis in 2019 [4]. Although POI is followed by some medical conditions such as genetic and autoimmune disorders, infections, chemotherapy, radiation and surgery but, the etiology is most often unknown [7]. This hypo estrogen- hyper gonadotropic disorder resulted to irregular menstruation, vasomotor symptoms, dyspareunia, infertility, osteoporosis, and endocrinological and cardiovascular diseases [8]. Both these short and long-term consequences have a endangerment to sexual and reproductive health (SRH) in women. Reproductive health contain physical, mental and social well-being in all matters relating to the reproductive system [9].

Studies have showed that physical and psychological effects of menopause affect women’s health and reduce their quality of life [10, 11]. Of course, the physical effects of hormone depletion in these women, such as osteoporosis, can depend on factors such as race, diet, and lifestyle [12]. Generally, the other studies founded one of the patients’ complaints was the adverse effects of POI on their general health [13–15].

These patients often have fertility problems and pregnancy is infrequent in these women, and egg donation is often the only solution for infertility [16]. many women diagnosed with POI find it difficult to accept their loss of fertility [17] and Infertility is one of the main causes of psychological, social, emotional problems in these women that even lead to divorce [18, 19]. However, in 5–10% of cases of POI pregnancy can occur spontaneously with hormone replacement therapy (HRT), in vitro fertilization (IVF), or, more recently, in-vitro maturation (IVM) or with stem cell therapy [16].

On the other hand, there are some studies that focused on nonphysical squeals of POI for instance anxiety, depression [20–23], impaired self-esteem [24–26] social and marital problems [14, 27–29]. Moreover, some researchers showed quality of life for women with POI is lower versus of the control group [10, 14, 30]. Also vaginal dryness and dyspareunia are commonly experienced by these women [14] and decreased women’s sexual health could negatively affect quality of life [31].

Considering that women with POI are in reproductive age and their fertility and sexual life are afflicted by this disorder directly it seems, that is necessary to evaluate sexual and reproductive health as a qualitative study in these women. Therefore, the present study aimed to investigate perception and experience of women with POI of sexual and reproductive health.

## Methods

This is a qualitative–descriptive study on perception and experience of Iranian women with POI of sexual and reproductive health.

### Participant recruitment

The participants were women with POI referred to the Avicenna fertility center, infertility clinic of the Valiasr Reproductive Health Research Center and, In-vitro fertilization department of Nikan hospital, who met the inclusion criteria.

The inclusion criteria were married women with POI based on the diagnostic criteria, being oriented and alert, being of Iranian nationality and Farsi speaking, being literate and lack of participation in qualitative interviews in the field of reproductive health.

The POI diagnostic criteria was based on European Society of Human Reproduction and Embryology (ESHRE) [2] guideline and included: experiencing amenorrhea lasting at least 4 months before the age of 40 accompanied with two FSH serum levels of more than 25 mIU/ml, and tested with at least a 1-month interval [2] which was subsequently confirmed by a gynecologist. After coordinating with the staff of the mentioned medical centers, and satisfying the women with POI by them, an appointment was made to interview these women in a secluded place in the clinic with the privacy of the participants and individually.

Purposive sampling was performed with a maximum variation of sampling in terms of age, education, the causes of POI, and parity and continued until data saturation i.e. until no new themes arise from further data collection [32].

### Interviews

To collect the information, we used semi-structured in-depth interviews. First, we explained the target of the research to the participants and informed them that the interviews would be recorded but at any time they prefer the audio recording would be cut off. Also they were assured that their personal information would be confidential. Afterward, the participants were asked about their personal information including age, education, occupation, menarche age, age of women at the time of the disorder, cause of POI, duration of the disease, family history of POI, having children, marital status (first–second), duration of marriage, number of pregnancies, number of children, number of unsuccessful pregnancies, and type of pregnancy (spontaneous-donated egg). Depended on the interview process, the sequence of questions was various for all participants. To deepen the interview and also to clarification the statements, we asked some questions for instance, “Would you explain in more detail?” and “What do you mean?” The interviews began in October 2019 and ended in May 2020, lasting between 40 and 100 min (mean 55 min).

### Ethical considerations

Ethical approval to conduct this study (IR.TUMS.FNM.REC.1398.018) was granted by the Ethics Committee of the School of Nursing and Midwifery, Tehran University of Medical Sciences, Tehran, Iran. At the beginning of the interview we explained the target of the research to the participants and informed them that the interviews would be recorded but at any time they prefer the audio recording would be cut off. Also they were assured that their personal information would be confidential. Informed consent was obtained from all individual participants.

### Data analyses

In current study, qualitative data were analyzed using conventional content analysis [33] based on the method proposed by Zhang and Wildmouth in eight steps as follows [34]: (1) Preparing data for qualitative content analysis; (2) decision on the unit of analysis; (3) categorize; (4) testing code on a sample of text; (5) extending the process of test coding to the entire text; (6) access to encoding stability; (7)conclusion from classified or coded data; and (8) reporting stage [34, 35].

To do so, in first step and after each interview, the recorded data were transcribed word by word. To get immersed in the data, that is necessary to read the transcriptions by the main researcher repeatedly. Additionally non-verbal messages participants such as tone, silence, crying and taking notes during the interview had been added to the text. At this stage, due to some incomplete explanations by the participants, the researcher realized that it is needed additional interviews with 4 participants. As a result, main researcher interviewed these participants again.

Second step contain extracting of initial codes from the meaning units (participants’ quotations). The rest of the steps are as follows: based on the similarities of these codes, subcategories were formed and finally they resulted in categories based on their relationship to each other. The researcher coded a sample of the text and the consistency of the data coding was monitored by two members of the research team. After agreement about the stability of coding, a repeatable process was obtained and expanded coding process to the entire text. The stability of coding (initial codes, subcategories and categories) was rechecked by two members of the research team and experts in the field of qualitative research. Categories were then tested on a wide range of data, in this way; from categories to sub categories and to related text and compared with each other. Data input and essence was extracted and it was examined whether the main categories were formed based on the essence of the data. Thus, the explanation of the main categories of reproductive-sexual health components in married women with premature ovarian failure was obtained and Max QDA version 12 software was used to manage the data in the qualitative phase of the research. The final stage consisted of reporting and interpreting the formed categories.

### Data trustworthiness

In present study, the credibility of the data was confirmed through purposeful sampling with maximum diversity, control of data by participants, review and revision of interviews, codes and categories extracted by members of the research team, long-term interaction with participants was conducted. To confirm dependability, the researcher provided the results of the research to an external observer to audit the research. Besides that, the same questions were asked of all participants so as not to threaten the risk of instability and inconsistency in research data collection.

Conformability was ensured using an external check. To establish transferability, the researcher tried to provide transferability by providing a clear description to the readers. Also, the sampling method of this study was purposeful, which helped to create transferability. On the other hand, maximum variation sampling was implemented [36, 37].

## Results

In this study, 16 women with POI, aged from 27 to 46 years old, and a POI duration of 1–25 years were interviewed. The age range of women at the time of POI and definitive diagnosis was 13 to 40 years. Among the participants, three women had remarried, two of whom had divorced after diagnosis POI due to infertility. The level of education of women was from primary to doctorate. The cause of the POI was mainly unknown, but in 2 participants, POI occurred after cancer treatment and a participant afflicted to POI following an autoimmune disease. The Other demographic characteristics of the participants are presented in Table 1.

**Table 1:** Demographic and reproductive characteristics of the participants

Characteristics	Mean (range)
Age (year)	38.06 (22–46)
Menarche age (year)	11.93 (11–17)
Age at the time of definitive diagnosis (year)	27.62 (13–40)
Disease duration (year)	8.5 (1–23)

Characteristics	Number
Education	
Primary	5
Diploma	5
Bachelor degree	2
Master’s degree	2
Ph.D. degree	2
Occupation	
Housewife	11
Employed	5
Number of marriages	
First	13
Second	3
Pregnancy history	
Yes	9
No	7
Live child	
Yes	5
No	11
Unsuccessful pregnancy history	
Yes	6
No	10
Type of pregnancy	
Natural	6
Donor egg	3
No have pregnancy	7
POI family history	
Yes	4
No	12
The cause of POI	
Idiopathic	13
Cancer treatment	2
Autoimmune disease	1

After content analysis of the interviews with a focus on the perception and experience of women with POI of reproductive-sexual health, four categories emerged (endangerment of women's health, psychological agitation, disruption of social life disturbance in sexual life), explained as follows.

### Endangerment of women's health

The results showed that all participants were concerned about the effects of decreased ovarian function and changes in hormone levels on their future health.

This main category consists of four subcategories (irregular menstruation, emergence of menopausal symptoms, infertility, signs of early aging) as follows:

### Irregular menstruation

Menstrual cycle changes (irregular menstrual cycle, primary amenorrhea or sudden cessation of menstrual bleeding) are one of the first suspicious signs of POI in women that resulted mostly to consult a physician.

One of the participants, who had POI for 8 years, said:

The first time my period became irregular, I went to the doctor and she told me that I should take hormone therapy. Before that, I had regular periods, but after 2-3 years, I did not have regular periods, and the doctor said there was a possibility of premature ovarian insufficiency (p. 9, 43 y).

Another participant who had regular periods for 27 years, stated:

Suddenly, I did not have another period. I went to the doctor. I had an ultrasound and found that I no longer had an ovum (p. 3, 46 y).

A number of participants did not experience menstruation at puberty and had primary amenorrhea, or spotted only once.

One participant that had a spontaneous POI, said*:*

I did not menstruate at all from the beginning, like my sister (p. 1, 30 y).

### Emergence of menopausal symptoms

Following changes in hormone levels, participants experienced some degree of menopausal complications.

One of the participants who had POI following treatment of cancer, said:

Dry uterus bothers me a lot, especially during sex (p. 10, 46 y).

Another participant who had POI for 10 years, stated:

It was very hard at first. In particular, flushing much annoyed me (p. 11, 44 y).

The other participant had POI with an autoimmune disease origin and had one live child with successful spontaneous pregnancy, said:

Premature ovarian insufficiency reduced libido (p. 8, 35 y).

### Infertility

This issue was the main concern of most participants and one of the main complaints of participants with POI was infertility.

A participant who had underwent chemotherapy for cancer treatment in 2008 and had lost her fertility for 11 years, said:

… I did not know before, but when I inclined to have a baby, I later realized that POI result to infertility (p. 2, 4 y).

Another woman who had divorced due to have a 17-year-old history of infertility and remarried, stated:

When I did ultrasound check for infertility, the report showed that my ovaries are very small like as ovaries in menopause women (p. 12, 43 y).

### Signs of early aging

Due to decreased levels of estrogen in afflicted women, some of them reported conditions like loss of beauty, wrinkling of the skin and decreased feeling of youth.

One participant, who had been suffering from premature ovarian failure since the age of 22 and for 10 years, said:

My first concern was this: … I was no longer beautiful (p. 16, 34 y).

The other participant that is pregnant currently with donated egg, said:

Eventually you f1eel the changes in your body. For example, you notice wrinkles on your skin (p. 9, 43 y).

One participant that had POI for 13 years, stated:

Although I am 37 years old, I do not feel young… I feel aging and I am old (p. 13, 38 y).

### Psychological agitation

POI occur in women is less than 40 years old, while the normal age of menopause in women is 45–55 years. Hence the acceptance of POI for participants was accompanied with psychological reactions.

This main category consists of three subcategories [anxiety reaction, mood reaction, agitation in the selection of childbearing] as following:

### Anxiety reaction

Participants experienced an onslaught of negative emotions after being diagnosed with POI by a physician, including feelings of despair, depression, a sense of aging, and shock from menopause.

A participant who had POI since the beginning of her marriage and for 5 years said:

When it told me to get menopause, I tried for traditional medicine but, due to that was not successful, I was disappointed (p. 7, 37 y).

Another participant expressed:

At that time, when I realized my problem, I became depressed and thought that I was the only one. It had a great effect on my mood (p. 1, 30 y).

A participant told *in despair:*

Because I don’t have children, I be early menopause,… that is, I got old…These are other signs of aging (p. 4, 46 y).

Another participant, who had POI since the age of 22 and had been struggling with it for 12 years, said:

I really didn’t expect such a thing at all. I was planning to have a planned pregnancy. But the exact opposite happened. The shock was so great it was the biggest shock of my life I have ever experienced (p. 16, 34 y).

### Mood reaction

Popular reactions in afflicted women with POI were included: feeling of uncertainty of future conditions, fear of disease outcome, feeling eternal problems [eternal infertility] negative effect on mood and weakness of the nerves.

One of the participants expressed with surprise and confusion:

I have no idea about the future. I'm very confused. I don’t know what will happen to me (p. 4, 42 y).

Also part of the conversation with a participant was as follows:

I think more about the fact that this [pregnancy] may never have happened to me (p. 14, 27 y).

Another participant said:

Premature ovarian insufficiency makes me angry quickly. I'll get mad soon (p. 10, 46 y).

A participant told:

I am worried that I will not have any problems after the age of 40. I am afraid of the consequences of this disease (p. 2, 34 y).

### Agitation in the selection of Childbearing

Considering that the options available to solve the problem of infertility in women with POI are currently limited and unfortunately there is no definitive treatment for female infertility in these women and the issue of cell therapy is being researched on animal models and do not use so far on humans, the only options offered to couples are the use of donated egg and adoption. Nevertheless, some participants opposed to accept them. If a participant commented on the issue of donated egg as follow:

“I think to myself about the baby… Because the egg is not mine, I am afraid I will not feel like a mother when she was born”*.* Also she continue:

I must convince myself about this pregnancy and deal with it (p. 15, 43 y).

Spiritual aspects of donated egg were important for some participants.

A participant was concerned about this, saying,

I do not care if I conceive with the donated egg, but its religious issue is important to me. It bothers me a little (p. 1, 30 y).

Moreover, it was important for a number of participants to know that the donor be a familiar person.

A participant stated:

I'm happy to have an ovum from my sister rather than a stranger (p. 2, 34 y).

### Disruption of social life

Most participants expressed POI has disrupted the social aspects of their lives. Social isolation, having privacy, unconscious jealousy and seeking support are four subcategories that related to this main category and be explain as follows:

### Social isolation

Patients stated that they were reluctant to be in public because of impatience, a tendency to be alone, and to become nervous about social relationships.

A participant said:

I'm not bored totally. I like to be at home, to be alone (p. 13, 38 y).

### Having privacy

Most afflicted women tended to maintain their privacy for fear of being judged by others, the importance of hiding the problem of infertility and believing in the privacy of the subject.

Some of the statements of the participants are as follows:

It is important for us that the donated egg is kept secret. Because if I get a donated egg, I will not be my own child and I will not judge (p. 6, 34 y).

This is a personal matter and has nothing to do with anyone (p. 13, 38 y).

### Unconscious jealousy

Some participants expressed a reluctance to associate with families that have children and they are jealous of pregnancies in others or seeing children.

If a participant that had POI for 26 years, said:

I was upset when I saw that others had children and became pregnant. Because I have a problem getting pregnant myself (p. 12, 43 y).

### Seeking support

This issue was the most important item that as a motivation factor helped afflicted women not only to accept complicated condition but also to pursue infertility treatment seriously. According to participants, the support of husbands, family and friends helped to increase hope and reduce psychological threat to women. In the meantime, the supportive role of the husbands was very prominent for women, as one of the participants that had POI for 18 years, said:

I am most supported by my husband. If he did not help me, I wouldn't be able to control the situation and control myself. He encourages me to continue my treatment and does not let me Disappointed. (p. 5, 30 y).

Another participant stated:

My sister, like me, had an early menopause. He tells me you are young now. Get treated sooner. You get the result. She is very hopeful and encourages me (p. 7, 37 y).

#### Disturbance in sexual life

In most patients, POI had a negative effect on the couple's sexual relationship.

Due to changes in hormone levels, women experienced sexual function disorders such as dyspareunia, reduced libido, and anorgasmia. These factors caused women to worry about the stability of their married life and the instability in marriage that they formed two subcategories from three.

In contrast, a number of other patients reported that POI had no effect on their sexuality.

The third subcategory was the ambivalence sensations that all of them explained as follows:

### Unstable marital life

The disease had a negative effect on sexual intercourse and sexual pleasure of affected women and on the other hand, sexual intercourse was important for the husband. As a result, a number of participants were concerned about the stability of married life.

A participant stated:

Before my problem, I had sexual desire, but now I do not have it at all, and this causes us to have sex more often with fights, and it has disrupted our relationship (p. 10, 46 y).

### Marital instability

Beside to decreased sexual satisfaction in couple, infertility also, leaded to some women felt insecure and worried about divorce. A few others threated to divorce from the spouse's family, and some be feared from their husband remarriage.

A participant said:

From the beginning of my marriage, I was stressed until now because I did not have children. My concern is to have children and that our marriage will fall apart (p. 1, 30 y).

Another participant stated:

Now my mother-in-law can easily divorce me. She says either bring a child or we will divorce you (p. 4, 42 y).

### The ambivalence sensations

The cessation of menstrual bleeding on the one hand created negative feelings for the participants and caused a kind of psychological pressure on them, but on the other hand had different effects on the participants’ spouses such as sexual satisfaction and helping to improve sexuality. Moreover, in the context of Iran religiously, having sex during a woman's period is against the Sharia, some patients even said that their partners were delighted with stopping in their menstruation to have sex freely. Therefore, these conditions caused women had been had a dual feeling about the negative impact of POI on their sexuality.

One of the participants said:

My husband says how good I am. I am comfortable without a condom. No man is happier than me (p. 5, 31 y).

Another participant, who has been suffering from POI since the age of 22 and for 12 years, said:

We are trying to cope with and we are trying to control and improve the condition ourselves. For example, we use lubricant for dyspareunia (p. 16, 34 y).

Or another participant said:

My husband thought POI meant we could no longer have sex. But when he saw that we had no problem with sex, he said it didn't matter. The important thing is that we can have sex without any limitation (p. 11, 44 y).

## Discussion

The present study implemented a qualitative approach to the evaluation of the SRH experiences of Iranian women with POI. In this study, we addressed POI is not just a medical issue. Unfortunately, in Iran, most medical staff pay attention to medical problems in afflicted women. However, due to important problems such as infertility and the early onset of menopausal symptoms at reproductive age, women are at risk for mental health problems and the instability of their sexual life. However, in order to provide better sexual and reproductive health services in these women, these items should be considered as much as the clinical and physical consequences of patients. This is the most important gap that is addressed in this study.

Our results showed that, one of the most common concerns of participants was changing in menstrual bleeding pattern and infertility that resulted to endangerment of women's health. While, This findings is similar to other researches [24, 38], founded afflicted women are concern about infertility and amenorrhea, in Bagheri et al. study sexual relationship was the most important concern of almost all participants [39]. Contrary to our study, Groff et al. [24] concluded that women generally had a good idea of their health. In our study, other endangerments to women's health, were early onset symptoms of menopause and aging in women. Similarly, Lockley et al. [38] in a hermeneutic phenomenology study with aimed to investigate the experience of spontaneous premature menopause for women and their partners, founded women were confused about feeling older and what will happen to their health in the long term. In line with our results, uncertain future is reported by the participants in another study. If they stated that their fertility and future were vague and their future was not clear [39]. Also some studies indicated that hot flushes and other symptoms of menopause were common complaint among the participants [13, 40, 41].

Psychological reactions of women with POI has been reported in several studies. The findings of Orshan et al. [29] in a phenomenology research on the lived experience of women with POI showed that women expressed depression, sadness and resignation. Also other researches demonstrated that women feelings at first hearing about the diagnosis of POI were confusion, depression, anxiety, emptiness, loss, shock, anger, denial, like our study [13, 24, 41, 42].

Benetti-pinto et al. in a case–control study demonstrated that having POI increases the risk of poor scores in physical and mental health [10]. In our study, participants also mentioned the physical and psychological effects of POI that finally are important for SRH.

The emotional reactions of Iranian women in our study were similar to those of Kuwaiti women, and included fear of not having children, anxiety, depression, unstable mood, aggression and irritability [43].

In a qualitative phenomenological study, the experience of infertile women in choosing to donate eggs is described [44]. These findings similar to our study pointed to the difficult acceptance of donated eggs and the importance of its religious dimensions.

In accordance with the other studies, we founded one of the POI consequences was social isolation. The results of studies showed some participants had a sense of not fitting in to a social group, or they were sometimes forced to leave or change their jobs due to constant questions [38, 39].

Having privacy was indicated in some studies such as ours. This includes the preferring of women with POI to conceal amenorrhea, infertility, early menopause and egg donation from the others. For case, most of them tended to keep the issue of egg donation as a family secret [44]. Besides, interviewees were suffered from being judged, being labeled and being blamed [41]. The stigma of donation, resulted in a negative attitude to them. So that hiding the disease became a major obsession to them [45].

The issue of social support of affected women has been addressed in several studies. Consistent with our study, in a study, the greatest source of women's emotional support was their husbands [24, 46] while Orshan founded one of the most common experiences of the women was a perceived lack of support from their health care providers regarding the symptoms that eventually led to a diagnosis of POI [29]. Unlike our findings, in a latest study, women complained for lack of supportive situation, which included lack of family support, lack of socio-economic support, and scattered information support [39].

Similar to our study, some Kuwaiti women with POI confessed to feeling resentful and jealousy of pregnant women [47] but, Iranian women said this feeling had limited their social relationships.

Impaired sexual relationship affected by the early onset of menopausal symptoms in women. Because vaginal dryness and decreased sex drive may have an adverse effect on the quality of sex and resulted in sexual dissatisfaction and finally, damage reproductive and sexual health. Kalantaridou et al. in a cross-sectional cohort study assessed sexual function in women with spontaneous POI. They resulted that the score of sexual function in women with POI was significantly lower than control women. Women with POI scored significantly lower on the sexual cognition, the orgasm and libido [48]. These findings are confirmed in other studies [49, 50].

Considering that, sexual relationship has an undeniable impact on married life cohesion and sustainability; it was one of the most important concerns of Iranian women in current interviews. Comparably, in a qualitative study, participants mentioned damaged sexual relationship and husband’s sexual dissatisfaction that resulted in threat married life [39].

Worry about possibility of divorce and loneliness has reported in Omu study as us and the POI group was significantly associated with divorce (*P* < 0.05) [43]. Moreover, the participants in our study had worry about husbands’ remarriage.

The ambivalence sensations in our study was related to create a negative feelings in women due to cessation of menstrual bleeding, on the other hand set a delighting and satisfaction their husbands of sexual intercourse. While, in Bagheri study, participants had dual emotions that related to women's optimism or pessimism about the success or failure of a donated egg pregnancy [39].

An important issue is to pay attention to the cultural-religious context and social conditions in each country and its effects on the SRH of these women. For example, in the present study, the experience of Iranian women with POI was examined. Due to cultural and religious reasons, only married women with official marriage included in this study, while a number of patients may have a sexual partner and have a different experience of SRH.

In Iran and most Arabic countries, the family is sacred in terms of religion and culture, and having children by married people is a value in that society. If a woman is unable to conceive, her social and sexual life as well as her mental health will suffer [35]. In other countries, however, POI may be a private matter and patients may be less likely to be judged by others [51]. As a result, SRH in women with POI is different depends on the sociocultural context in each country [52].

### The strength of this study

In recent years the issue of POI has been considered by researchers and studies have been conducted to investigate the consequences of POI on quality of life, mental and sexual health of women but, until now has not been performed a comprehensive study to assessment the women’s SRH. As a result, the strength of this study is the assessing of reproductive and sexual health experience of women with POI simultaneously and in one study.

### Suggestion

Studies showed that the perception and experience of afflicted women in physical and mental health aspects are similar in different races and countries while, the social and sexual dimensions of the issue are influenced by the cultural and religious context in each country. As a result, it is recommended that further qualitative studies be conducted with this purpose in other countries around the world, and with different religions. It is also suggested that based on such studies, a reproductive and sexual health care package be designed specifically for women with POI to reduce patients’ concerns that help to increase their peace of mind and rehabilitation.

## Conclusion

We concluded that POI affects different aspects of SRH (women physical, psychological, social and sexual heath) and in addition, it is revealed that POI make the ambivalence sensations in women that the one hand create negative feelings for the women and cause a kind of psychological pressure on them, but on the other hand has different effects on the participants’ husbands such as sexual satisfaction and helping to improve sex relationship. Therefore, knowledge of their concerns by health professionals is helpful to improve service delivery and increasing the effectiveness of treatment interventions by a comprehensive health care attitude.

## Data Availability

Data sharing is not applicable to this article as all transcripts and recordings were deleted permanently following data analysis to protect the identity and privacy of the participants.
